# PAC-Bayes Unleashed: Generalisation Bounds with Unbounded Losses

**DOI:** 10.3390/e23101330

**Published:** 2021-10-12

**Authors:** Maxime Haddouche, Benjamin Guedj, Omar Rivasplata, John Shawe-Taylor

**Affiliations:** 1 ENS Paris-Saclay, 91190 Gif-sur-Yvette, France; maxime.haddouche@ens-paris-saclay.fr; 2 Centre for Artificial Intelligence, Department of Computer Science, University College London, London WC1V 6LJ, UK; o.rivasplata@cs.ucl.ac.uk (O.R.); j.shawe-taylor@ucl.ac.uk (J.S.-T.); 3 Inria, Lille–Nord Europe Research Centre and Inria London Programme, 59800 Lille, France

**Keywords:** statistical learning theory, PAC-Bayes, generalisation bounds

## Abstract

We present new PAC-Bayesian generalisation bounds for learning problems with unbounded loss functions. This extends the relevance and applicability of the PAC-Bayes learning framework, where most of the existing literature focuses on supervised learning problems with a bounded loss function (typically assumed to take values in the interval [0;1]). In order to relax this classical assumption, we propose to allow the range of the loss to depend on each predictor. This relaxation is captured by our new notion of *HYPothesis-dependent rangE* (HYPE). Based on this, we derive a novel PAC-Bayesian generalisation bound for unbounded loss functions, and we instantiate it on a linear regression problem. To make our theory usable by the largest audience possible, we include discussions on actual computation, practicality and limitations of our assumptions.

## 1. Introduction

Since its emergence in the late 1990s, the PAC-Bayes theory (see the seminal works of [1,2,3], the recent survey by [4] and work by [5]) has been a powerful tool to obtain generalisation bounds and to derive efficient learning algorithms. Generalisation bounds are helpful for understanding how a learning algorithm may perform on future similar batches of data. While the classical generalization bounds typically address the performance of individual predictors from a given hypothesis class, PAC-Bayes bounds typically address a randomized predictor defined by a distribution over the hypothesis class.

PAC-Bayes bounds were originally meant for binary classification problems [6,7,8], but the literature now includes many contributions involving any bounded loss function (without loss of generality, with values in [0;1]), not just the binary loss. Our goal is to provide new PAC-Bayes bounds that are valid for unbounded loss functions, and thus extend the usability of PAC-Bayes to a much larger class of learning problems. To do so, we reformulate the general PAC-Bayes theorem of [9] and use it as basic building block to derive our new PAC-Bayes bound.

Some ways to circumvent the bounded range assumption on the losses have been explored in the recent literature. For instance, one approach consists of assuming a tail decay rate on the loss, such as sub-gaussian or sub-exponential tails [10,11]; however, this approach requires the knowledge of additional parameters. Some other works have also looked into the analysis for heavy-tailed losses, e.g., ref. [12] proposed a polynomial moment-dependent bound with *f*-divergences, while [13] devised an exponential bound that assumes the second (uncentered) moment of the loss is bounded by a constant (with a truncated risk estimator, as recalled in Section 4 below). A somewhat related approach was explored by [14], who do not assume boundedness of the loss, but instead control higher-order moments of the generalization gap through the Efron-Stein variance proxy. See also [5].

We investigate a different route here. We introduce the *HYPothesis-dependent rangE* (HYPE) condition, which means that the loss is upper-bounded by a term that depends on the chosen predictor (but does not depend on the data). Thus, effectively, the loss may have an arbitrarily large range. The HYPE condition allows us to derive an upper bound on the exponential moment of a suitably chosen functional, which, combined with the general PAC-Bayes theorem, leads to our new PAC-Bayes bound. To illustrate it, we instantiate the new bound on a linear regression problem, which additionally serves the purpose of illustrating that our HYPE condition is easy to verify in practice, given an explicit formulation of the loss function. In particular, we shall see in the linear regression setting that a mere use of the triangle inequality is enough to check the HYPE condition. The technical assumptions on which our results are based are comparable to those of the classical PAC-Bayes bounds; we state them in full detail, with discussions, for the sake of clarity and to make our work accessible.

**Our contributions are twofold.** (i) We propose PAC-Bayesian bounds holding with unbounded loss functions, therefore overcoming a limitation of the mainstream PAC-Bayesian literature for which a bounded loss is usually assumed. (ii) We analyse the bound, its implications, limitations of our assumptions, and their usability by practitioners. We hope this will extend the PAC-Bayes framework into a widely usable tool for a significantly wider range of problems, such as unbounded regression or reinforcement learning problems with unbounded rewards.

**Outline.**Section 2 introduces our notation and definition of the HYPE condition and provides a general PAC-Bayesian bound, which is valid for any learning problem complying with a mild assumption. For the sake of completeness, we present how our approach (designed for the unbounded case) behaves in the bounded case (Section 3). This section is not the core of our work, but rather serves as a safety check and particularises our bound to more classical PAC-Bayesian assumptions. We also provide numerical experiments. Section 4 introduces the notion of *softening functions* and particularises Section 2’s PAC-Bayesian bound. In particular, we make explicit all terms in the right-hand side. Section 5.1 extends our results to linear regression (which has been studied from the perspective of PAC-Bayes in the literature, most recently by [15]). We also experimentally illustrate the behaviour of our bound. Finally, Section 6 presents, in detail, related works and Section 7 contains all proofs of the original claims we make in the paper.

## 2. Framework and Preliminary Results

The learning problem is specified by three variables (H,Z,ℓ) consisting of a set H of predictors, the data space Z, and a loss function $\ell :H\times Z\to R^{+}$.

For a given positive integer *m*, we consider size-*m* datasets. The space of all possible datasets of this fixed size is $S=Z^{m}$; an arbitrary element of this space is $s=(z_{1},\ldots ,z_{m})$. We denote *S* as a random dataset: $S=(Z_{1},\ldots ,Z_{m})$ where the random data points $Z_{i}$ are independent and sampled from the same distribution μ over Z. We call μ the data-generating distribution. The assumption that the $Z_{i}$’s are *independent and identically distributed* is typically called the i.i.d. data assumption. It means that the random sample *S* (of size *m*) has distribution $\mu^{⊗m}$ which is the product of *m* copies of μ.

For any predictor h∈H, we define the *empirical risk* of *h* over a sample *s*, denoted $R_{s}\left(h\right)$, and the *theoretical risk* of *h*, denoted R(h), as:

$$R_{s}\left(h\right)=\frac{1}{m}\sum_{i=1}^{m}\ell \left(h,z_{i}\right)\;\mathrm{and}\;R\left(h\right)=E_{\mu }\left[\ell \left(h,Z\right)\right]$$

respectively, where $E_{\mu }\left[\ell \left(h,Z\right)\right]$ denotes the expectation with respect to Z∼μ. Finally, we define the *risk gap* $\Delta_{s}\left(h\right)=R\left(h\right)-R_{s}\left(h\right)$ for any h∈H and s∈S. Often, $\Delta_{s}\left(h\right)$ is referred to as the generalisation gap.

Notice that for a random dataset *S*, the empirical risk $R_{S}\left(h\right)$ is random, with expected value $E_{\mu^{⊗m}}\left[R_{S}\left(h\right)\right]=R\left(h\right)$, where $E_{\mu^{⊗m}}$ the expectation under the distribution of the random sample *S*.

In general, $E_{\mu }\left[\cdot \right]$ denotes an expectation under the distribution μ. When we want to emphasize the role of the random variable Z∼μ we write $E_{Z}\left[\cdot \right]$ or $E_{Z∼\mu }\left[\cdot \right]$ instead of $E_{\mu }\left[\cdot \right]$. We use a similar convention for expectations related to any other distributions and random quantities. We now introduce the key concept to our analysis.

**Definition** **1.** (HYPE). *A loss function $\ell :H\times Z\to R^{+}$ is said to satisfy the **hypothesis-dependent range** (HYPE) condition if there exists a function $K:H\to R^{+}\backslash \left\{0\right\}$ such that $\sup_{z\in Z}\ell \left(h,z\right)\leq K\left(h\right)$ for every predictor h. We then say that ℓ is HYPE(K) compliant.*

Let $M_{1}^{+}\left(H\right)$ be the set of probability distributions on H. We assume that all considered probability measures on H are defined on a fixed σ-algebra over H, while the notation $M_{1}^{+}\left(H\right)$ hides the σ-algebra, for simplicity. For $P,P^{\prime }\in M_{1}^{+}\left(H\right)$, the notation $P^{\prime }\ll P$ indicates that $P^{\prime }$ is absolutely continuous with respect to *P* (i.e., $P^{\prime }\left(A\right)=0$ if P(A)=0 for measurable A⊂H). We write $P^{\prime }∼P$ to indicate that $P^{\prime }\ll P$ and $P\ll P^{\prime }$, i.e., these two distributions are absolutely continuous with respect to each other.

We now recall a result from Germain et al. [9]. Note that while implicit in many PAC-Bayes works (including theirs), we make it explicit that both the prior *P* and the posterior *Q* must be absolutely continuous with respect to each other. We discuss this restriction below.

**Theorem** **1.** (Adapted from [9], Theorem 2.1.) *For any $P\in M_{1}^{+}\left(H\right)$ with no dependency on data, for any function $F:R^{+}\times R^{+}\to R$, define the exponential moment:*

$$\chi :=E_{S}E_{h∼P}\;\left[e^{F(R_{S}\left(h\right),R\left(h\right))}\right]\;.$$

*If F is convex, then for any δ∈[0;1], with probability of at least 1−δ over random samples S, simultaneously for all $Q\in M_{1}^{+}\left(H\right)$ such that Q∼P we have:*

$$\begin{array}{c} F\left(E_{h∼Q}\left[R_{S}\left(h\right)\right],E_{h∼Q}\left[R(h)\right]\right)\leq \mathrm{KL}\left(Q||P\right)+\log\left(\frac{\chi }{\delta }\right). \end{array}$$

The proof is deferred to Section 7.1. Note that the proof in [9] requires that P≪Q, although it is not explicitly stated; we highlight this in our own proof. While Q≪P is classical and necessary for the KL(Q||P) to be meaningful, P≪Q appears to be more restrictive. In particular, we have to choose *Q* such that it has the exact same support as *P* (e.g., choosing a Gaussian and a truncated Gaussian is not possible). However, we can still apply our theorem when *P* and *Q* belong to the same parametric family of distributions, e.g., both ‘full-support’ Gaussian or Laplace distributions, but these are just two examples and there are many others.

Note that Alquier et al. [10] (Theorem 4.1) adapted a result from Catoni [8], which only requires Q≪P. This comes at the expense of what Alquier et al. [10] (Definition 2.3) called a *Hoeffding’s assumption*, which means that the exponential moment χ is assumed to be bounded by a function depending only on the hyperparameters (such as the dataset size *m* or parameters given by Hoeffding’s assumption). Our analysis does not require this assumption, which might prove restrictive in practice.

Theorem 1 may be seen as a basis to recover many classical PAC-Bayesian bounds. For instance, $F\left(x,y\right)=2m{\left(x-y\right)}^{2}$, recovers McAllester’s bound as recalled in [4] (Theorem 1). To get a usable bound, the outstanding task is to bound the exponential moment χ. Note that a previous attempt has been made in [11], as described in Section 6.1 below. Furthermore, under the assumption that the distribution *P* has no dependency on the data, we may swap the order of integration in the exponential moment thanks to Fubini-Tonelli’s theorem and the positiveness of the exponential:

$$\chi =E_{h∼P}E_{S}\left[e^{F(R_{S}\left(h\right),R\left(h\right))}\right]\;.$$

This is the starting point for the way that the exponential moment was handled in several works in the PAC-Bayes literature. Essentially, for a fixed *h*, one may upper-bound the innermost expectation (with respect to *S*) using standard exponential moment inequalities.

In this work, we will use Theorem 1 with $F\left(x,y\right)=m^{\alpha }D\left(x,y\right)$, where α>0, and $D:R^{+}\times R^{+}\to R$ is a convex function. In this case, the high-probability inequality of the theorem takes the form:

(1) $$\begin{array}{c} D\left(E_{h∼Q}\left[R_{S}\left(h\right)\right],E_{h∼Q}\left[R(h)\right]\right)\leq \\ \;\;\;\;\;\;\;\;\;\;\;\frac{1}{m^{\alpha }}\left(\mathrm{KL}\left(Q||P\right)+\log\left(\frac{1}{\delta }E_{h∼P}E_{S}\;e^{m^{\alpha }D\left(R_{S}\left(h\right),R\left(h\right)\right)}\right)\right). \end{array}$$

Our goal is to control $E_{S}\;e^{m^{\alpha }D\left(R_{S}\left(h\right),R\left(h\right)\right)}$ for a fixed *h*, when D(x,y)=y−x. This will readily give us control on the exponential moment χ. To do so, we propose the following theorem:

**Theorem** **2.** *Let h∈H be a fixed predictor and α∈R. If the loss function ℓ is HYPE(K) compliant, then for $\Delta_{S}\left(h\right)=R\left(h\right)-R_{S}\left(h\right)$ we have:*

$$E_{S}\left[e^{m^{\alpha }\Delta_{S}\left(h\right)}\right]\leq \exp\left(\frac{K{\left(h\right)}^{2}}{2m^{1-2\alpha }}\right).$$

**Proof.**  Let h∈H. Then:

$$\begin{array}{cc} E_{S}\left[e^{m^{\alpha }\Delta_{S}\left(h\right)}\right]\; & =E\left[\exp\left(m^{\alpha -1}\sum_{i=1}^{m}\left(l\left(h,Z_{i}\right)-R\left(h\right)\right)\right)\right]\\ \; & =E\left[\prod_{i=1}^{m}\exp\left(m^{\alpha -1}\left(\ell \left(h,Z_{i}\right)-R\left(h\right)\right)\right)\right]\\ \; & =\prod_{i=1}^{m}E\left[\exp\left(m^{\alpha -1}\left(\ell \left(h,Z_{i}\right)-R\left(h\right)\right)\right)\right]. \end{array}$$

We now apply Hoeffding’s lemma, for any i∈{1..m}, the random (in $Z_{i}$) variable $\ell \left(h,Z_{i}\right)-R\left(h\right)$ is centered, taking values in [−K(h);K(h)], so that:

$$E\left[\exp\left(m^{\alpha -1}\left(\ell \left(h,Z_{i}\right)-R\left(h\right)\right)\right)\right]\leq \exp\left(m^{2\alpha -2}\frac{4K{\left(h\right)}^{2}}{8}\right)$$

and finally:

$$E_{S}\left[e^{m^{\alpha }\Delta_{S}\left(h\right)}\right]\leq \prod_{i=1}^{m}\exp\left(m^{2\alpha -2}\frac{4K{\left(h\right)}^{2}}{8}\right)=\exp\left(\frac{K{\left(h\right)}^{2}}{2m^{1-2\alpha }}\right).$$

 □

The strength of this result lies in the fact that $\frac{K{\left(h\right)}^{2}}{m^{1-2\alpha }}$, is a decreasing factor in *m*, when α≤1/2, and more generally, one can control how fast the exponential moment will explode when *m* grows by the choice of the hyperparameter α.

For convenient cross-referencing, we state the following rewriting of Theorem 1.

**Theorem** **3.** *Let the loss ℓ be HYPE(K) compliant. For any $P\in M_{1}^{+}\left(H\right)$ with no data dependency, for any α∈R and for any δ∈[0;1], with probability of at least 1−δ over size-m random samples S, simultaneously for all Q such that Q∼P we have:*

$$E_{h∼Q}\left[R(h)\right]\leq E_{h∼Q}\left[R_{S}\left(h\right)\right]+\frac{1}{m^{\alpha }}\left(\mathrm{KL}\left(Q||P\right)+\log\frac{E_{h∼P}\left[\exp\left(\frac{K{\left(h\right)}^{2}}{2m^{1-2\alpha }}\right)\right]}{\delta }\right).$$

**Proof.**  We first apply Theorem 1 with $F\left(x,y\right)=m^{\alpha }\left(y-x\right)$. More precisely, we use Equation (1) with D(x,y)=y−x. We then conclude with Theorem 2.  □

## 3. Safety Check: The Bounded Loss Case

### 3.1. Theoretical Results

At this stage, the reader might wonder whether this new approach allows for the recovery of known results in the bounded case: the answer is yes.

In this section, we study the case where *ℓ* is bounded by some constant $C\in R^{+}\backslash \left\{0\right\}$. In other words, we consider the case that $\sup_{h}\sup_{z}\ell \left(h,z\right)\leq C$. We provide a bound, valid for any choice of “priors” *P* and “posteriors” *Q* such that P∼Q, which is an immediate corollary of Theorem 3.

**Proposition** **1.** *Let ℓ be HYPE(K) compliant, with K(h)=C constant, and let α∈R. Let $P\in M_{1}^{+}\left(H\right)$ be a distribution with no data dependency. Then, for any δ∈[0;1], with probability of at least 1−δ over random m-samples S, simultaneously for all $Q\in M_{1}^{+}\left(H\right)$ such that Q∼P we have:*

$$E_{h∼Q}\left[R(h)\right]\leq E_{h∼Q}\left[R_{S}\left(h\right)\right]+\frac{\mathrm{KL}(Q||P)+\log(1/\delta )}{m^{\alpha }}+\frac{C^{2}}{2m^{1-\alpha }}.$$

**Remark** **1.** *We provide Proposition 1 to evaluate the robustness of our approach. For instance, by comparing it with the PAC-Bayesian bound found in Germain et al. [11]. This discussion can be found in Section 6.1, where the bound from Germain et al. [11] is presented in detail.*

**Remark** **2.** *At first glance, a naive remark: in order to control the rate of convergence of all the terms of the bound in Proposition 1 (as is often the case in classical PAC-Bayesian bounds), then the only case of interest is in fact $\alpha =\frac{1}{2}$. However, one could notice that the factor $C^{2}$ is not optimisable, while the KL is. In this way, if it appears that $C^{2}$ is too big, in practice, one wants to have the ability to attenuate its influence as much as possible and this may lead us to consider α<1/2. The following lemma answers this question.*

**Lemma** **1.** *For any given $K_{1}>0$, the function $f_{K_{1}}\left(\alpha \right):=\frac{K_{1}}{m^{\alpha }}+\frac{C^{2}}{m^{1-\alpha }}$ reaches its minimum at*

$$\alpha_{0}=\frac{1}{2}+\frac{1}{2\log(m)}\log\left(\frac{2K_{1}}{C^{2}}\right).$$

**Proof.**  The explicit calculus of the $f_{K_{1}}^{{}^{\prime }}$ and the resolution of $f_{K_{1}}^{{}^{\prime }}\left(\alpha \right)=0$ provides the result.  □

**Remark** **3.** *Lemma 1 indicates that with a fixed “prior” P and “posterior” Q, taking $K_{1}=\mathrm{KL}\left(Q||P\right)+\log\left(1/\delta \right)$, gives the optimised value of the bound in Proposition 1. We numerically show in Section 3.2 (first experiment there) that optimising α leads to significantly better results.*

Now the only remaining question is how to optimise the KL divergence. To do so, we may need to fix an “informed prior” to minimise the KL divergence with an interesting posterior. This idea has been studied by [16,17] and, more recently, by Mhammedi et al. [18], Rivasplata et al. [5], among others. We will adapt it to our problem in the simplest way.

We now introduce some additional notation. For a sample $s=(z_{1},\ldots ,z_{m})$ and k∈{1..m}, we define $s_{\leq k}:=\left\{z_{1},\ldots ,z_{k}\right\}$ and $s_{>k}:=\left\{z_{k+1},\ldots ,z_{m}\right\}$. Then, similarly, for a random sample *S*, we have the splits $S_{\leq k}$ and $S_{>k}$.

**Proposition** **2.** *Let ℓ be HYPE(K) compliant, with constant K(h)=C, and $\alpha_{1},\alpha_{2}\in R$. Consider any “priors” $P_{1}\in M_{1}^{+}\left(H\right)$ (possibly dependent on $S_{>m/2}$) and $P_{2}\in M_{1}^{+}\left(H\right)$ (possibly dependent on $S_{\leq m/2}$). Then, for any δ∈[0;1], with probability of at least 1−δ over random size-m samples S, simultaneously for all $Q\in M_{1}^{+}\left(H\right)$ such that $Q∼P_{1}$ and $Q∼P_{2}$ we have:*

$$\begin{array}{c} E_{h∼Q}\left[R(h)\right]\leq E_{h∼Q}\left[R_{S}\left(h\right)\right]+\frac{1}{2}\left(\frac{\mathrm{KL}(Q||P_{1})+\log\left(2/\delta \right)}{{\left(m/2\right)}^{\alpha_{1}}}+\frac{C^{2}}{2{\left(m/2\right)}^{1-\alpha_{1}}}\right)\\ \;\;\;\;\;+\frac{1}{2}\left(\frac{\mathrm{KL}(Q||P_{2})+\log\left(2/\delta \right)}{{\left(m/2\right)}^{\alpha_{2}}}+\frac{C^{2}}{2{\left(m/2\right)}^{1-\alpha_{2}}}\right). \end{array}$$

**Proof.**  Let $P_{1},P_{2},Q$ be as stated in Proposition 2. We first notice that by using Proposition 1 on the two halves of the sample, we obtain, with a probability of at least 1−δ/2:

$$E_{h∼Q}\left[R(h)\right]\leq E_{h∼Q}\left[\frac{1}{m/2}\sum_{i=1}^{m/2}\ell \left(h,Z_{i}\right)\right]+\frac{\mathrm{KL}(Q||P_{1})+\log\left(2/\delta \right)}{{\left(m/2\right)}^{\alpha_{1}}}+\frac{C^{2}}{2{\left(m/2\right)}^{1-\alpha_{1}}}$$

and also with probability at least 1−δ/2:

$$E_{h∼Q}\left[R(h)\right]\leq E_{h∼Q}\left[\frac{1}{m/2}\sum_{i=1}^{m/2}\ell \left(h,Z_{m/2+i}\right)\right]+\frac{\mathrm{KL}(Q||P_{2})+\log\left(2/\delta \right)}{{\left(m/2\right)}^{\alpha_{2}}}+\frac{C^{2}}{2{\left(m/2\right)}^{1-\alpha_{2}}}.$$

Hence, with a probability of at least 1−δ, both inequalities hold, and the result follows by adding them and dividing by 2. □

**Remark** **4.** *One can notice that the main difference between Proposition 2 and Proposition 1 lies in the implicit PAC-Bayesian paradigm that our priors must not depend on the data. With this last proposition, we implicitly allow $P_{1}$ to depend on $S_{>m/2}$ and $P_{2}$ on $S_{\leq m/2}$, which can in practice lead to far more accurate priors. We numerically show this fact in Section 3.2’s second experiment. Note that this idea is not new and has been studied, for instance, in [19] for the specific case of SVMs.*

### 3.2. Numerical Experiments

Our experimental framework has been inspired by the work of [18].

**Settings.** We generate synthetic data for classification, and we are using the 0–1 loss. The data space is $Z=X\times Y=R^{d}\times \left\{0,1\right\}$ with d∈N. The set of predictors H is parameterised with *d*-dimensional ‘weight’ vectors: $H=\{h_{w}:X\to Y\;|\;w\in R^{d}\}$. For simplicity, we identify $h_{w}$ with *w* and we also identify the space H, with the weight space $W=R^{d}$. For z=(x,y)∈Z and w∈W, we define the loss as $\ell \left(w,z\right):=|𝟙\left\{\phi (w^{⊤}x)>1/2\right\}-y|$, where $\phi \left(r\right)=\frac{1}{1+e^{-r}}$. We want to learn an optimised predictor given a dataset $S={\left(Z_{i}\right)}_{i=1..m}$ where $Z_{i}=\left(X_{i},Y_{i}\right)$. To do so, we use *regularised logistic regression* and compute:

(2) $$\begin{array}{c} \hat{w}\left(S\right):=\arg\min_{w\in W}\lambda \frac{{\left||w|\right|}^{2}}{2}-\frac{1}{m}\sum_{i=1}^{m}y_{i}\log\left(\phi (w^{⊤}x_{i})\right)+\left(1-y_{i}\right)\log\left(1-\phi (w^{⊤}x_{i})\right) \end{array}$$

where λ is a fixed regularisation parameter.

We also restrict the probability distributions (over $W=R^{d}$), considered for this learning problem. We consider the Gaussian distribution $N(w,\sigma^{2}I_{d})$ with centre $w\in R^{d}$ and diagonal covariance $\sigma^{2}I_{d}\in R^{d\times d}$ with $\sigma^{2}>0$.

**Parameters.** We set δ=0.05,λ=0.01. We approximately solve Equation (2) by using the minimize function of the optimisation module in Python, with the Powell method. To approximate gaussian expectations, we use Monte-Carlo sampling.

**Synthetic data.** We generate synthetic data for d=10 according to the following process: for a fixed sample size *m*, we draw $X_{1},\ldots ,X_{m}$ under the multivariate Gaussian distribution $N(0,I_{d})$ and for each *i* we compute the label if $X_{i}$ as: $Y_{i}=𝟙\left\{\phi \left(w^{*⊤}x_{i}\right)>1/2\right\}$ where $w^{*}$ is the vector formed by the *d* first digits of the number π.

**Normalisation trick.** Given the predictors shape, we notice that for any w∈W:

$$𝟙\left\{\phi \left(w^{⊤}x\right)>1/2\right\}=1\;\Leftrightarrow \;\frac{1}{1+\exp(-w^{⊤}x)}>\frac{1}{2}\;\Leftrightarrow \;w^{⊤}x<0.$$

Thus, the value of the prediction is exclusively determined by the sign of the inner product, and this quantity is definitely not influenced by the norm of the vector. Then, for any sample *S*, we call the **normalisation trick** the fact of considering $\hat{w}\left(S\right)/\left|\right|\hat{w}\left(S\right)\left|\right|$ instead of $\hat{w}\left(S\right)$ in our calculations. This process will not deteriorate the quality of the prediction and will considerably enhance the value of the KL divergence.

#### 3.2.1. First Experiment

**Figure 1:**
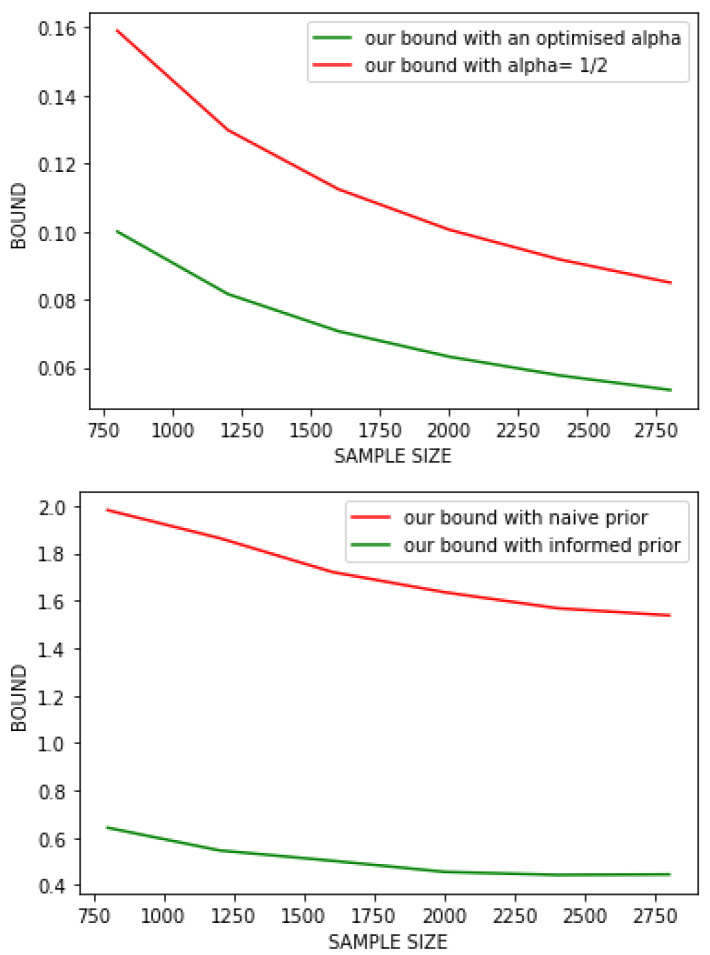
Above, result of the first experiment which highlight the importance of optimising α. Below, result of the second experiment which show how effective an informed prior is.

Our goal here is to highlight the point discussed in Remark 2, e.g., the influence of the parameter α in Proposition 1. We arbitrarily fix $\sigma_{0}^{2}=1/2$, and define our *naive prior* as $P_{0}=N\left(0,\sigma_{0}^{2}I_{d}\right)$. For a fixed dataset *S*, we define our posterior as $P\left(S\right):=N(\hat{h}\left(S\right),\sigma^{2}I_{d})$, with $\sigma^{2}\in \left\{1/2,\ldots ,1/2^{J}\right\}$ (for $J=\log_{2}\left(m\right)$) such that it is minimising the bound among candidates. We computed two curves: first, Proposition 1 with α=1/2 second, Proposition 1 again with α equals to the value proposed in Lemma 1. Notice that to compute this last bound, we first optimised our choice of posterior with α=1/2 and then optimised α, to be consistent with Lemma 1. Indeed, we proved this lemma by assuming that the KL divergence was already fixed, hence our optimisation process is in two steps. Note that we chose to apply the normalisation trick here, we then obtained the left curve of Figure 1.

**Discussion.** From this curve, we formulate several remarks. First, we remark on this specific case, our theorem provides a tight result in practice (with an error rate lesser than 10% for the bound with optimised alpha). Second, we can now confirm that choosing an optimised α leads to a tighter bound. In further studies, it will be relevant to adjust α with regards to the different terms of our bound instead of looking for an identical convergence rate for all terms.

#### 3.2.2. Second Experiment

We now study Proposition 2 to see if an informed prior effectively provides a tighter bound than a naive one. We will use the notations introduced in Proposition 2. For a dataset *S*, we define $w_{1}\left(S\right)=w\left(S_{>m/2}\right)$ as the vector resulting from the optimisation of Equation (2) on $S_{>m/2}$. Similarly, we define $w_{2}\left(S\right):=w\left(S_{\leq m/2}\right)$. We arbitrarily fix $\sigma_{0}^{2}=1/2$, and define our *informed priors* as: $P_{1}=N\left(w_{1}\left(S\right),\sigma_{0}^{2}I_{d}\right)$ and $P_{2}=N\left(w_{2}\left(S\right),\sigma_{0}^{2}I_{d}\right)$. Finally, we define our posterior as $P\left(S\right):=N(\hat{w}\left(S\right),\sigma^{2}I_{d})$, with $\sigma^{2}\in \left\{1/2,\ldots ,1/2^{J}\right\}$ (for $J=\log_{2}\left(m\right)$) with $\sigma^{2}$ optimising the bound among the same candidate than the first experiment. We computed two curves: first, Proposition 1 with α optimised accordingly to Lemma 1 secondly, Proposition 2 with $\alpha_{1},\alpha_{2}$ optimised as well, and informed priors as defined above. We chose to not apply the normalisation trick here, we then obtained the right curve of Figure 1.

**Discussion.** It is clear, that with this framework, having an informed prior is a powerful tool to enhance the quality of our bound. Notice that we voluntarily chose to not apply the normalisation trick here. The reason is that this trick appears to be too powerful in practice, and applying it leads to counterproductive results; to highlight our point: the bound without informed prior would be tighter than the one with informed prior. Furthermore, this trick is linked to the specific structure of our problem and is not valid for any classification problem. Thus, the idea of providing informed priors remains an interesting tool for most cases.

## 4. PAC Bayesian Bounds with Smoothed Estimator

We now move on to control the right-hand side term in Theorem 3 when *K* is not constant. A first step is to consider a transformed estimate of the risk, inspired by the truncated estimator from [20], also used in [21], and more recently in [13]. The following is inspired by the results of [13], which we summarise in Section 6.

The idea is to modify the estimator $R_{S}\left(h\right)$ for any *h* by introducing a threshold *t* and a function ψ which will attenuate the influence of the empirical losses ${\left(\ell \left(h,Z_{i}\right)\right)}_{i=1..m}$ that exceed *t*.

**Definition** **2.** ψ-risks. *For every t>0, $\psi :R^{+}\to R^{+}$, for any h∈H, we define the empirical ψ-risk $R_{S,\psi ,t}$ and the theoretical ψ-risk $R_{\psi ,t}$ as follows:*

$$R_{S,\psi ,t}\left(h\right):=\frac{t}{m}\sum_{i=1}^{m}\psi \left(\frac{\ell (h,Z_{i})}{t}\right)\;\mathrm{and}\;R_{\psi ,t}\left(h\right)=E_{\mu }\left[t\;\psi \left(\frac{\ell (h,Z)}{t}\right)\right]$$

*where Z∼μ. Notice that $E_{S}\left[R_{S,\psi ,t}\left(h\right)\right]=R_{\psi ,t}\left(h\right)$.*

We now focus on what we call *softening functions*, i.e., functions that will temper high values of the loss function *ℓ*.

**Definition** **3.** (Softening function). *We say that $\psi :R^{+}\to R^{+}$ is a softening function if:*

- *∀x∈[0;1],ψ(x)=x,*
- *ψ is non-decreasing,*
- *∀x≥1,ψ(x)≤x.*

*We let F denote the set of all softening functions.*

**Remark** **5.** *Notice that those three assumptions ensure that ψ is continuous at* 1. *For instance, the functions f:x↦x𝟙{x≤1}+𝟙{x>1} and $g:x\mapsto x𝟙\left\{x\leq 1\right\}+\left(2\sqrt{x}-1\right)𝟙\left\{x>1\right\}$ are in F. In Section 6 we compare these softening functions and those used by Holland [13].*

Using ψ∈F, for a fixed threshold t>0, the softened loss function $t\psi \left(\frac{\ell (h,z)}{t}\right)$ verifies for any h∈H, z∈Z:

$$t\;\psi \left(\frac{\ell (h,z)}{t}\right)\leq t\;\psi \left(\frac{K(h)}{t}\right)$$

because ψ is non-decreasing. In this way, the exponential moment in Theorem 3 can be far more controllable. The trade-off lies in the fact that softening *ℓ* (instead of taking directly *ℓ*) will deteriorate our ability to distinguish between two bad predictions when both of them are greater than *t*. For instance, if we choose ψ∈F such as ψ=1 on [1;+∞) and t>0, if $\psi \left(\ell (h,z)/t\right)=1$ for a certain pair (h,z), then we cannot tell how far ℓ(h,z) is from *t* and we only can affirm that ℓ(h,z)≥t.

We now move on to the following lemma, which controls the shortfall between $E_{h∼Q}\left[R\left(h\right)\right]$ and $E_{h∼Q}\left[R_{\psi ,t}\left(h\right)\right]$ for all $Q\in M_{1}^{+}\left(H\right)$, for a given ψ and t>0. To do that, we assume that *K* admits a finite moment under any posterior distribution:

(3) $$\begin{array}{c} \forall Q\in M_{1}^{+}\left(H\right),\;E_{h∼Q}\left[K\left(h\right)\right]<+\infty . \end{array}$$

For instance, in the case of H identified with a weight space $W=R^{N}$, and if *K* is polynomial in ||w|| (where ||.|| denotes the Euclidean norm), then this assumption holds if we consider Gaussian priors and posteriors.

**Lemma** **2.** *Assume that Equation (3) holds, and let ψ∈F, $Q\in M_{1}^{+}\left(H\right),t>0$. We have:*

$$E_{h∼Q}\left[R\left(h\right)\right]\leq E_{h∼Q}\left[R_{\psi ,t}\left(h\right)\right]+E_{h∼Q}\left[K\left(h\right)𝟙\left\{K(h)\geq t\right\}\right].$$

**Proof.**  Let ψ∈F, $Q\in M_{1}^{+}\left(H\right),t>0$. We have, for h∈H:

$$\begin{array}{cc} \; & R\left(h\right)-R_{\psi ,t}\left(h\right)\\ \; & \;=E_{Z∼\mu }\left[\ell \left(h,Z\right)-t\psi \left(\frac{\ell (h,Z)}{t}\right)\right] \end{array}$$

and using that ∀x∈[0,1],ψ(x)=x,

$$\begin{array}{c} \;=E_{Z∼\mu }\left[\left(\ell \left(h,Z\right)-t\psi \left(\frac{\ell (h,Z)}{t}\right)\right)𝟙\left\{\ell \left(h,Z\right)\geq t\right\}\right] \end{array}$$

while using that ℓ(h,z)≤K(h),

$$\begin{array}{c} \;=E_{Z∼\mu }\left[\left(\ell \left(h,Z\right)-t\psi \left(\frac{\ell (h,Z)}{t}\right)\right)𝟙\left\{\ell \left(h,Z\right)\geq t\right\}𝟙\left\{K(h)\geq t\right\}\right] \end{array}$$

and continuing:

$$\begin{array}{cc} \;\leq E_{Z∼\mu }\left[\ell (h,Z)𝟙\{\ell (h,Z)\geq t\}\right]𝟙\left\{K(h)\geq t\right\} & \;\;\;\;\;\;\;\;\;(\psi \geq 0) \end{array}$$

$$\begin{array}{cc} \;\leq K\left(h\right)P_{Z∼\mu }\left\{\ell (h,Z)\geq t\right\}𝟙\left\{K(h)\geq t\right\} & \;\;\;\;\;\;\;(\ell (h,Z)\leq K(h)) \end{array}$$

Finally, by crudely bounding the probability by 1, we get:

$$R\left(h\right)\leq R_{\psi ,t}\left(h\right)+K\left(h\right)𝟙\left\{K(h)\geq t\right\}.$$

Hence the result by integrating over H with respect to *Q*. □

Finally we present the following theorem, which provides a PAC-Bayesian inequality bounding the theoretical risk by the empirical ψ-risk for ψ∈F.

**Theorem** **4.** *Let ℓ be HYPE(K) compliant, and assume K satisfies Equation (3). Then for any $P\in M_{1}^{+}\left(H\right)$ with no data dependency, for any α∈R, for any ψ∈F and for any δ∈[0;1], with probability of at least 1−δ over size-m random samples S, simultaneously for all Q such that Q∼P we have:*

$$\begin{array}{cc} E_{h∼Q}\left[R(h)\right]\; & \leq E_{h∼Q}\left[R_{S,\psi ,t}\left(h\right)\right]+E_{h∼Q}\left[K(h)𝟙\{K(h)\geq t\}\right]\\ \; & \;+\frac{\mathrm{KL}\left(Q||P\right)+\log\left(\frac{1}{\delta }\right)}{m^{\alpha }}\\ \; & \;+\frac{1}{m^{\alpha }}\log\left(E_{h∼P}\left[\exp\left(\frac{t^{2}}{2m^{1-2\alpha }}\psi {\left(\frac{K(h)}{t}\right)}^{2}\right)\right]\right). \end{array}$$

**Proof.**  Let ψ∈F, we define the ψ-loss:

$$\ell_{2}\left(h,z\right)=t\psi \left(\frac{\ell (h,z)}{t}\right).$$

Since ψ is non decreasing, we have for all (h,z)∈H×Z:

$$\ell_{2}\left(h,z\right)\leq t\psi \left(\frac{K(h)}{t}\right):=K_{2}\left(h\right).$$

Thus, we apply Theorem 3 to the learning problem defined with $\ell_{2}$: for any α and δ∈(0,1), with probability at least 1−δ over size-*m* random samples *S*, simultaneously for all *Q* such that Q∼P we have:

$$\begin{array}{cc} E_{h∼Q}\left[R_{\psi ,t}\left(h\right)\right] & \leq E_{h∼Q}\left[R_{S,\psi ,t}\left(h\right)\right]+\frac{\mathrm{KL}\left(Q||P\right)+\log\left(\frac{1}{\delta }\right)}{m^{\alpha }}\\ \; & \;+\frac{1}{m^{\alpha }}\log\left(E_{h∼P}\left[\exp\left(\frac{K_{2}{\left(h\right)}^{2}}{2m^{1-2\alpha }}\right)\right]\right). \end{array}$$

We then add $E_{h∼Q}\left[K\left(h\right)𝟙\left\{K(h)\geq t\right\}\right]$ on both sides of the latter inequality and apply Lemma 2. □

**Remark** **6.** *Notice that the function ψ:x↦x𝟙{x≤1}+𝟙{x>1} is such that for any given prior P we have $E_{h∼P}\left[\exp\left(\frac{t^{2}}{2m^{1-2\alpha }}\psi {\left(\frac{K(h)}{t}\right)}^{2}\right)\right]<+\infty$. So the exponential moment can be controlled with a good choice of ψ. Thus the strength of Theorem 4 is to provide a PAC-Bayesian bound valid for any set of posterior measures verifying Equation (3). The choice of ψ minimising the bound is still an open problem.*

## 5. The Linear Regression Problem

### 5.1. Theoretical Result

We now focus on the celebrated linear regression problem and see how our theory translates to that particular learning problem. We assume that the data is a size-*m* random sample $S={\left(Z_{i}\right)}_{i=1..m}$ where the $Z_{i}$ are i.i.d. drawn from the distribution μ, and $Z_{i}=\left(X_{i},Y_{i}\right)$ with $X_{i}\in R^{N}$, $Y_{i}\in R$.

Our goal here is to find the most accurate predictor $h_{w}$ (with $w\in R^{N}$), with respect to the loss function $\ell \left(h_{w},z\right)=\left|\langle w,x\rangle -y\right|$, where z=(x,y). We will make the following mild assumption: there exists B,C∈R+\{0} such that for all z=(x,y) drawn under μ:

||x||≤Band|y|≤C

where ||.|| is the norm associated to the classical inner product of $R^{N}$. Under this assumption we note that for all z=(x,y) drawn according to μ, we have:

$$\begin{array}{c} \ell \left(h_{w},z\right)=|\langle w,x\rangle -y|\leq |\langle w,x\rangle \left|+|y\right]\leq \left||w||.||x||+|y\right|\leq B||w||+C. \end{array}$$

Thus we define $K(h_{w})=B||w||+C$ for $w\in R^{N}$. If we first restrict ourselves to the framework of Section 2, we want to use Theorem 3 and doing so, our goal is to bound $\xi :=E_{w∼P}\left[\exp\left(\frac{K{\left(w\right)}^{2}}{2m^{1-2\alpha }}\right)\right]$. The shape of *K* invites us to consider a Gaussian prior. Indeed, we notice that if $P=N(0,\sigma^{2}I_{N})$ with $0<\sigma^{2}<\frac{m^{1-2\alpha }}{B^{2}}$, then ξ<+∞. Notice that we cannot take just any Gaussian prior, however with a small α, the condition $0<\sigma^{2}<\frac{m^{1-2\alpha }}{B^{2}}$ may become quite loose. Thus, we have the following:

**Theorem** **5.** *Let α∈R and N≥6. Assume that the loss ℓ is HYPE(K) compliant with K(h)=B||h||+C, with B>0,C≥0. For a prior distribution, consider any Gaussian $P=N(0,\sigma^{2}I_{N})$ with $\sigma^{2}=t\frac{m^{1-2\alpha }}{B^{2}}$, 0<t<1. Then, for any δ∈[0;1], with probability of at least 1−δ over size-m random samples S, simultaneously for all $Q\in M_{1}^{+}\left(H\right)$ such that P∼Q we have:*

$$\begin{array}{cc} E_{h∼Q}\left[R\left(h\right)\right] & \leq E_{h∼Q}\left[R_{S}\left(h\right)\right]+\frac{\mathrm{KL}\left(Q||P\right)+\log\left(2/\delta \right)}{m^{\alpha }}+\frac{C^{2}}{2m^{1-\alpha }}\left(1+f{\left(t\right)}^{-1}\right)\\ \; & \;+\frac{N}{m^{\alpha }}\left(\log\left(1+\left(\frac{C}{\sqrt{2f\left(t\right)m^{1-2\alpha }}}\right)\right)+\log\left(\frac{1}{\sqrt{1-t}}\right)\right) \end{array}$$

*where $f\left(t\right)=\frac{1-t}{t}$.*

The proof is deferred to Section 7.2. To compare our result with those found in the literature, we can fix α=1/2. Doing so, we lose the dependency in *m* for the choice of the variance of the prior (which now only depends on *B*), but we recover the classic decreasing factor $1/\sqrt{m}$.

**Remark** **7.** *Notice that for now we did not use Section 4, even if we could (because K is polynomial in ||w|| and we consider Gaussian priors and posteriors, so Equation (3) is satisfied). Doing so, we obtained a bound which appears to depend linearly on the dimension N. In practice, N may be too big, and in this case, introducing an adapted softening function ψ (one can think for instance of ψ(x)=x𝟙{x≤1}+𝟙{x>1}) is a powerful tool to attenuate the weight of the exponential moment. This also extends the class of authorised Gaussian priors by avoidance, to stick with a variance $\sigma^{2}=t\frac{m^{1-2\alpha }}{B^{2}}$,  0<t<1.*

### 5.2. Numerical Experiment

#### 5.2.1. Setting

In this section we apply Theorem 5 on a concrete linear regression problem. The situation is as follows: we want to approximate the function $f\left(x\right)=\sqrt{\langle w^{*},x\rangle }$, where $w^{*}\in R^{d}$. We assume that $W={\left[-c,c\right]}^{d}$ so that $w^{*}$ lies in an hypercube centred at 0 of half-side c>0, i.e., the set $\left\{{\left(w_{i}\right)}_{i=1,\ldots ,d}|\forall i,|w_{i}|\leq c\right\}$. Doing so we have $\left|\right|w^{*}\left|\right|\leq c\sqrt{d}$.

Furthermore, we assume that input data are drawn inside a hypercube of half-side e>0, i.e., $X={\left[-e,e\right]}^{d}$. Doing so we have for any data $x,||x||\leq e\sqrt{d}$.

For any data $x\in R^{d}$, we define y=f(x). As before, we identify the hypothesis set H with the weight space $W=R^{d}$. As described in Section 5.1, we set $\ell \left(h_{w},x,y\right)=\left|\langle w,x\rangle -y\right|$. We then remark that for any (w,x,y):

$$\begin{array}{cc} \ell (h_{w},x,y) & \leq \left|\langle w,x\rangle |+|y\right|\leq \left||w||||x||+\right|\sqrt{\langle w^{*},x\rangle }|\\ \; & \leq e\sqrt{d}\left||w|\right|+\sqrt{\left|\right|w^{*}\left||.||x|\right|}\leq e\sqrt{d}\left||w|\right|+\sqrt{c\sqrt{d}.e\sqrt{d}}\\ \; & \leq e\sqrt{d}\left||w|\right|+\sqrt{cde}. \end{array}$$

Then we can define $B=e\sqrt{d}$ and $C=\sqrt{cde}$ to apply Theorem 5. We restrict (as before) the class of distributions over W to be *d*-dimensional Gaussians:

$$\left\{N\left(w,\sigma^{2}I_{d}\right)|w\in H,\sigma^{2}\in R^{+}\right\},$$

which is the set of candidate distributions for this learning problem. Recall that in practice, given a fixed α∈R, we are only allowed to consider priors such that their variance $\sigma^{2}\in ]0;\frac{m^{1-2\alpha }}{B^{2}}[$. We want to learn an optimised predictor (posterior) given a random dataset $S={\left(\left(X_{i},Y_{i}\right)\right)}_{i=1,\ldots ,m}$. To do so, we consider synthetic data.

#### 5.2.2. Synthetic Data

We draw $w^{*}$ under a Gaussian (with mean 0 and standard deviation equal to 5) truncated to the hypercube centered at 0 of the half-side c>0. We generate synthetic data according to the following process: for a fixed sample size *m*, we draw $X_{1},\ldots ,X_{m}$ under a Gaussian (with mean 0 and standard deviation equal to 5) truncated to the hypercube centered at 0 of the half-side e>0.

#### 5.2.3. Experiment

**Figure 2:**
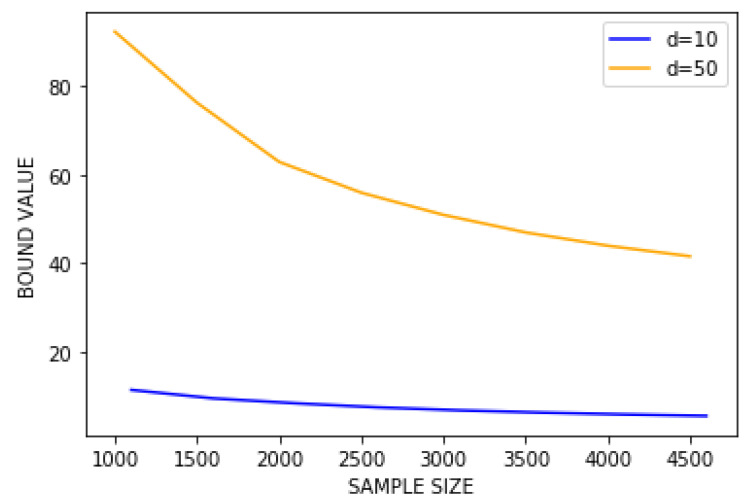
Evaluation of the right hand side in Theorem 5 with d=10 and d=50.

First, we fix c=e=10. Our goal here is to obtain a generalisation bound on our problem. We fix arbitrarily, for a fixed α∈R, $t_{0}=1/2$ and $\sigma_{0}^{2}=t_{0}\frac{m^{1-2\alpha }}{B^{2}}$ and we define our *naive prior* as $P_{0}=N\left(0,\sigma_{0}^{2}I_{d}\right)$. For a given dataset *S*, we define our posterior as $Q\left(S\right):=N(\hat{w}\left(S\right),\sigma^{2}I_{d})$, with $\sigma^{2}\in \left\{\sigma_{0}^{2}/2,\ldots ,\sigma_{0}^{2}/2^{J}\right\}$ ($J=\log_{2}\left(m\right)$), such that it is minimising the bound among candidates. Note that all the previously defined parameters are dependent on α, which is why we choose α∈{i/step∣0≤i≤step} for step a fixed integer (in practice step = 8 or 16) and we take the value of α minimising the bound among the candidates as well. Figure 2 contains two figures, one with d=10, the other with d=50. On each figure are computed the right-hand side term in 5 with an optimised α for each step.

#### 5.2.4. Discussion

To the the best of our knowledge, this is the first attempt to numerically compute PAC-Bayes bounds for unbounded problems, making it impossible to compare to other results. We stress, however, that obtaining numerical values for the bound without assuming a bounded loss is a significant first step. Furthermore, we consider a rather hard problem: *f* is not linear, so we cannot rely on a linear approximation fitting perfectly data, and the larger the dimension, the larger the error, as illustrated by Figure 2. Thus, for any posterior *Q*, the quantity $E_{h∼Q}\left[R\left(h\right)\right]$ is potentially large in practice and our bound might not be tight. Finally, notice that optimising α (instead of taking α=1/2 to recover a classic convergence rate) leads to a significantly better bound. A numerical example of this assertion is presented in Section 3.2. We aim to conduct further studies to consider the convergence rate as an hyperparameter to optimise, rather than selecting the same rate for all terms in the bound.

## 6. Existing Work

### 6.1. Germain et al., 2016

In Germain et al. [11] (Section 4), a PAC-Bayesian bound has been provided for all *sub-gamma* losses with a variance $t^{2}$ and scale parameter c>0, under a data distribution μ and a prior *P*, i.e., losses such that for every $\lambda \in \left(0,\frac{1}{c}\right)$ the following is satisfied:

$$\log\left(\frac{1}{\delta }E_{h∼P}E_{S}\;e^{\lambda (R\left(h\right)-R_{S}\left(h\right))}\right)\leq \frac{t^{2}}{c^{2}}\left(-\log\left(1-c\lambda \right)-\lambda c\right)\leq \frac{\lambda^{2}t^{2}}{2(1-c\lambda )}.$$

Note that a sub-gamma loss (with regards to μ and *P*) is potentially unbounded. Germain et al. then propose the following PAC-Bayesian bound:

**Theorem** **6.** *Ref. [11]. If the loss ℓ is sub-gamma with a variance $t^{2}$ and scale parameter c, under the data distribution μ and a fixed prior P∈H, then for any δ∈[0;1], with probability 1−δ over size-m random samples, simultaneously for all Q≪P we have:*

$$E_{h∼Q}\left[R(h)\right]\leq E_{h∼Q}\left[R_{S}\left(h\right)\right]+\frac{\mathrm{KL}(Q||P)+\log(1/\delta )}{m}+\frac{t^{2}}{2(1-c)}.$$

Theorem 6 will be quoted several times in this paper given that it is a concrete PAC Bayesian bound provided with the will to overcome the constraint of a bounded loss. It is also one of the only one found in the literature.

Can we apply this theorem to the bounded case? The answer is yes: we remark that thanks to Hoeffding’s lemma, if *ℓ* is bounded by C>0, then for any h∈H it holds that $R_{S}\left(h\right)-R\left(h\right)\in \left[-C,C\right]$ almost surely. So, ∀λ∈R, $\log E_{z∼\mu }\left[e^{\lambda (R\left(h\right)-R_{S}\left(h\right)}\right]\leq \frac{\lambda^{2}C^{2}}{2}$. Therefore, for any prior *P*, we have:

$$\log E_{h∼P}E_{z∼\mu }\left[e^{\lambda (R\left(h\right)-R_{S}\left(h\right)}\right]\leq \frac{\lambda^{2}C^{2}}{2}\;.$$

Thus, *ℓ* is sub-gamma with variance $C^{2}$ and scale parameter 0. Then, Theorem 6 can be applied with $t^{2}=C^{2}$, c=0.

**Comparison with Proposition 1.** We remark that by taking K=C and α=1 in Proposition 1, we are recovering Theorem 6. However, our approach allows us to say that if we can obtain a more precise form of *K* such that ∀h∈H, K(h)≤C and *K* is non-constant, 3, will ensure that:

$$\frac{1}{m^{\alpha }}\log\left(E_{h∼P}\left[\exp\left(\frac{K{\left(h\right)}^{2}}{2m^{1-2\alpha }}\right)\right]\right)\leq \frac{C^{2}}{2m^{1-\alpha }}.$$

Thus, having precise information on the behavior of the loss function *ℓ*, with regards to the predictor *h*, allows us to obtain a tighter control of the exponential moment, and hence a tighter bound.

**Remark** **8.** *We can see that Theorem 6 cannot control the factor $C^{2}/2$. However, Ref. [11] remarked on this apparent weakness and partially corrected this issue [11] (Section 4, Equations (13) and (14)). Indeed, they proposed to balance the influence of m between the different terms of the PAC-Bayes bound by providing the same convergence rate in $1/\sqrt{m}$ to all terms.*

*We can then see Proposition 1 as a proper generalisation of Germain et al. [11] (Section 4, Equations (13) and (14)). Indeed, our bound exhibits properly the influence of the parameter α. Thus, we understand (and Lemma 1 proves it) that the choice of α deserves a study in itself in the way it is now a parameter of our optimisation problem. This fact has already been highlighted in Alquier et al. [10] (Theorem 4.1) (where $\lambda :=m^{\alpha }$).*

### 6.2. Holland, 2019

In [13], Holland proposed a PAC Bayesian inequality with unbounded loss. For that, he introduced a function ψ verifying a few specific conditions, different to those used in Section 4 to define our set of softening functions. Indeed, he considered a function ψ such that:

- ψ is bounded,
- ψ is non decreasing,
- it exists b>0 such that for all u∈R:
  (4) $$\begin{array}{c} -\log\left(1-u+\frac{u^{2}}{b}\right)\leq \psi \left(u\right)\leq \log\left(1+u+\frac{u^{2}}{b}\right). \end{array}$$

We remark that, as Holland did, we supposed that our softening functions are non-decreasing. We chose softening functions to be equal to the identity function (x↦x) on [0,1], which is quite restrictive. However, we are imposing softening functions to be lesser than the identity on $\left[1,+\infty \right)$; whereas, Holland supposed ψ to be bounded and satisfy Equation (4). A concrete example of such a function ψ, lies in the piecewise polynomial function of Catoni and Giulini [21], defined by:

$$\psi \left(u\right)=\left\{\begin{array}{cc} -2\sqrt{2}/3 & \;\mathrm{if}\;u\leq -\sqrt{2}\\ u-u^{3}/6 & \;\mathrm{if}\;u\in [-2\sqrt{2}/3,2\sqrt{2}/3]\\ 2\sqrt{2}/3 & \mathrm{otherwise}. \end{array}\right.$$

As in Section 4, we are considering the ψ-empirical risk $R_{S,\psi ,t}$ for any t>0. Holland provided his theorem given the fact the following assumptions are realised:

- Bounds on lower-order moments. For all h∈H, we have $E_{Z∼\mu }\left[\ell {\left(h,Z\right)}^{2}\right]\leq M_{2}<+\infty$ and $E_{Z∼\mu }\left[\ell {\left(h,Z\right)}^{3}\right]\leq M_{3}<+\infty$.
- Bounds on the risk. For all h∈H, we suppose $R\left(h\right)\leq \sqrt{mM_{2}/(4\log\left(\delta^{-1}\right)}$.
- Large enough confidence, we require $\delta \leq e^{-1/9}$.

Now we can state Holland’s theorem.

**Theorem** **7.** *Ref. [13]. Let P be a prior distribution on model H. Let the three assumptions listed above hold. Setting $t^{2}=mM_{2}/\left(2\log\left(\delta^{-1}\right)\right)$, then for any δ∈[0;1], with probability of at least 1−δ over the random draw of the size-m sample S, simultaneously for all Q it holds that:*

$$\begin{array}{cc} E_{h∼Q}\left[R(h)\right] & \leq E_{h∼Q}\left[R_{S,\psi ,t}\left(h\right)\right]+\frac{1}{\sqrt{m}}\left(\mathrm{KL}\left(Q||P\right)+\frac{1}{2}\log\left(\frac{8\pi M_{2}}{\delta^{2}}\right)-1\right)\\ \; & +\frac{1}{\sqrt{m}}\nu^{*}\left(H\right)+O\left(\frac{1}{m}\right) \end{array}$$

*where:*

$$\nu^{*}\left(H\right):=\frac{E_{h∼P}\left[\exp\left(\sqrt{m}\left(R\left(h\right)-R_{S,\psi ,t}\left(h\right)\right)\right)\right]}{\;E_{h∼P}\left[\exp\left(R\left(h\right)-R_{S,\psi ,t}\left(h\right)\right)\right]}.$$

## 7. Proofs

### 7.1. Proof of Theorem 1

**Proof.**  Let $F:R^{+}\times R^{+}\mapsto R$ be a convex function, *P* a fixed prior, and δ∈[0,1]. Since $E_{h∼P}\left[e^{F(R_{S}\left(h\right),R\left(h\right))}\right]$ is a nonnegative random variable, we know that, by Markov’s inequality, for any h∈H:

$$P\left(E_{h∼P}\left[e^{F(R_{S}\left(h\right),R\left(h\right))}\right]>\frac{1}{\delta }E_{S}\;E_{h∼P}\left[e^{F(R_{S}\left(h\right),R\left(h\right))}\right]\right)\leq \delta .$$

So with probability of at least 1−δ, we have:

$$E_{h∼P}\left[e^{F(R_{S}\left(h\right),R\left(h\right))}\right]\leq \frac{1}{\delta }E_{S}\;E_{h∼P}\left[e^{F(R_{S}\left(h\right),R\left(h\right))}\right]=\frac{\chi }{\delta }.$$

Applying the log function on each side of this inequality gives us with probability of at least 1−δ over samples *S*:

$$\log\left(E_{h∼P}\left[e^{F(R_{S}\left(h\right),R\left(h\right))}\right]\right)\leq \log\left(\frac{\chi }{\delta }\right).$$

We now rename $A:=\log\left(E_{h∼P}\left[e^{F(R_{S}\left(h\right),R\left(h\right))}\right]\right)$.

Furthermore, if we denote by $\frac{dQ}{dP}$ the Radon-Nikodym derivative of *Q* with respect to *P* when Q≪P, we then have, for all *Q* such that Q∼P:

$$\begin{array}{ccc} A\; & =\log\left(E_{h∼Q}\left[\frac{dP}{dQ}e^{F(R_{S}\left(h\right),R\left(h\right))}\right]\right)\\ \; & =\log\left(E_{h∼Q}\left[{\left(\frac{dQ}{dP}\right)}^{-1}e^{F(R_{S}\left(h\right),R\left(h\right))}\right]\right) & \;\;\;\;(\frac{dP}{dQ}={\left(\frac{dQ}{dP}\right)}^{-1}) \end{array}$$

and by concavity of log and Jensen’s inequality,

$$\begin{array}{c} \geq -E_{h∼Q}\left[\log\left(\frac{dQ}{dP}\right)\right]+E_{h∼Q}\left[F(R_{S}\left(h\right),R\left(h\right))\right]\\ =-\mathrm{KL}\left(Q||P\right)+E_{h∼Q}\left[F(R_{S}\left(h\right),R\left(h\right))\right] \end{array}$$

while by convexity of *F* with Jensen’s inequality,

$$\begin{array}{c} \geq -\mathrm{KL}\left(Q||P\right)+F\left(E_{h∼Q}\left[R_{S}\left(h\right)\right],E_{h∼Q}\left[R(h)\right]\right). \end{array}$$

Hence, for *Q* such that Q∼P,

$$F\left(E_{h∼Q}\left[R_{S}\left(h\right)\right],E_{h∼Q}\left[R(h)\right]\right)\;\leq \;\mathrm{KL}(Q||P)+A.$$

So with probability 1−δ, for *Q* such that Q∼P,

$$\begin{array}{c} F\left(E_{h∼Q}\left[R_{S}\left(h\right)\right],E_{h∼Q}\left[R(h)\right]\right)\;\leq \;\mathrm{KL}\left(Q||P\right)+\log\left(\frac{\chi }{\delta }\right). \end{array}$$

This completes the proof of Theorem 1. □

### 7.2. Proof of Theorem 5

We first provide a technical property. Recall that:

$$\xi =E_{h∼P}\left[\exp\left(\frac{K{\left(h\right)}^{2}}{2m^{1-2\alpha }}\right)\right].$$

**Proposition** **3.** *Let α∈R. Suppose the loss ℓ is HYPE(K) compliant with K(h)=B||h||+C, with B>0, C≥0. Then, for any Gaussian prior $P=N(0,\sigma^{2}I_{N})$ with $\sigma^{2}=t\frac{m^{1-2\alpha }}{B^{2}}$, 0<t<1 and N≥6 we have:*

$$\xi \leq 2\exp\left(\frac{C^{2}}{2m^{1-2\alpha }f\left(t\right)}\left(1+f(t)\right)\right)\frac{1}{{\left(\sqrt{1-t}\right)}^{N}}{\left(1+\left(\frac{C}{\sqrt{2f\left(t\right)m^{1-2\alpha }}}\right)\right)}^{N-1}$$

*with $f\left(t\right)=\frac{1-t}{t}$.*

**Proof.**  We recall that $\sigma^{2}=t\frac{m^{1-2\alpha }}{B^{2}}$. By expliciting the expectation and K(h) we thus obtain:

$$\begin{array}{ccc} \xi \; & ={\left(\frac{1}{\sqrt{2\pi \sigma^{2}}}\right)}^{N}\int_{h\in R^{N}}\exp\left(\frac{{\left(B||h||+C\right)}^{2}}{2m^{1-2\alpha }}-\frac{{\left||h|\right|}^{2}B^{2}}{2tm^{1-2\alpha }}\right)dh\; & \\ \; & ={\left(\frac{1}{\sqrt{2\pi \sigma^{2}}}\right)}^{N}\int_{h\in R^{N}}\exp\left(-\frac{1}{2m^{1-2\alpha }}\left(f\left(t\right)B^{2}{\left||h|\right|}^{2}-2BC||h||-C^{2}\right)\right)dh\; & \\ \; & ={\left(\frac{1}{\sqrt{2\pi \sigma^{2}}}\right)}^{N}\int_{h\in R^{N}}\exp\left(-\frac{B^{2}f\left(t\right)}{2m^{1-2\alpha }}\left({\left||h|\right|}^{2}-\frac{2C||h||}{Bf(t)}-\frac{C^{2}}{B^{2}f\left(t\right)}\right)\right)dh\; & \\ \; & =\exp\left(\frac{C^{2}}{2m^{1-2\alpha }f\left(t\right)}\left(1+f(t)\right)\right)\frac{1}{{\left(\sqrt{2\pi \sigma^{2}}\right)}^{N}}\int_{h\in R^{N}}\exp\left(-\frac{B^{2}f\left(t\right)}{2m^{1-2\alpha }}{\left(\left||h|\right|-\frac{C}{Bf(t)}\right)}^{2}\right)dh. \end{array}$$

We will use the spherical coordinates in *N*-dimensional Euclidean space given in [22]:

$$\varphi :\left(h_{1},\ldots ,h_{N}\right)\to \left(r,\varphi_{1},\ldots ,\varphi_{N-1}\right)$$

where especially r=||h|| and also the Jacobian of ϕ is given by:

$$d^{N}V=r^{N-1}\prod_{k=1}^{N-2}\sin^{k}\left(\varphi_{N-1-k}\right)=r^{N-1}d_{S^{N-1}}V.$$

Let us also precise that as given in Blumenson [22] (page 66), we have that the surface of the sphere of radius 1 in *N*-dimensional space is:

$$\int_{\varphi_{1},\ldots ,\varphi_{N-1}}d_{S^{N-1}}V\;d\varphi_{1}\ldots d\varphi_{N-1}=\frac{2{\sqrt{\pi }}^{N}}{\Gamma \left(\frac{N}{2}\right)}$$

where Γ is the Gamma function defined as:

$$\Gamma \left(x\right)=\int_{0}^{+\infty }t^{x-1}e^{-t}dt\;\mathrm{for}\;x>-1.$$

Then, if we set:

$$A:=\int_{h\in R^{N}}\exp\left(-\frac{B^{2}f\left(t\right)}{2m^{1-2\alpha }}{\left(\left||h|\right|-\frac{C}{Bf(t)}\right)}^{2}\right)dh$$

we obtain by a change of variable:

A=∫r,φ1,…,φN−1exp−B2f(t)2m1−2αr−CBf(t)2dNVdrdφ1…dφN−1=2πNΓN2∫r=0+∞exp−B2f(t)2m1−2αr−CBf(t)2rN−1dr=2πNΓN2∫r=−CBf(t)+∞r+CBf(t)N−1exp−B2f(t)2m1−2αr2dr=2πNΓN2∑k=0N−1N−1kCBf(t)N−k−1∫r=−CBf(t)+∞rkexp−B2f(t)2m1−2αr2dr.

We fix a random variable *X* such that:

$$X∼N\left(0,\frac{m^{1-2\alpha }}{B^{2}(f\left(t\right)}\right).$$

We then have for any *k* positive integer, if *k* is even:

$$\begin{array}{cc} \int_{r=-\frac{C}{Bf(t)}}^{+\infty }r^{k}\exp\left(-\frac{B^{2}f\left(t\right)}{2m^{1-2\alpha }}r^{2}\right)dr\; & \leq \int_{r=-\infty }^{+\infty }r^{k}\exp\left(-\frac{B^{2}f\left(t\right)}{2m^{1-2\alpha }}r^{2}\right)dr\\ \; & \leq \sqrt{2\pi \frac{m^{1-2\alpha }}{B^{2}f\left(t\right)}}{E[|X|}^{k}]. \end{array}$$

And if *k* is odd:

$$\begin{array}{cc} \int_{r=-\frac{C}{Bf(t)}}^{+\infty }r^{k}\exp\left(-\frac{B^{2}f\left(t\right)}{2m^{1-2\alpha }}r^{2}\right)dr\; & \leq \int_{r=0}^{+\infty }r^{k}\exp\left(-\frac{B^{2}f\left(t\right)}{2m^{1-2\alpha }}r^{2}\right)dr\\ \; & \leq \sqrt{2\pi \frac{m^{1-2\alpha }}{B^{2}f\left(t\right)}}{E[|X|}^{k}𝟙\left(X\geq 0\right)]\\ \; & \leq \sqrt{2\pi \frac{m^{1-2\alpha }}{B^{2}f\left(t\right)}}{E[|X|}^{k}]. \end{array}$$

So we have:

A≤2πNΓN2∑k=0N−1N−1kCBf(t)N−k−12πm1−2αB2f(t)E[|X|k].

As precised in [23], we have for any *k*:

$${E[|X|}^{k}]={\left(\sqrt{\frac{m^{1-2\alpha }}{B^{2}f\left(t\right)}}\right)}^{k}2^{k/2}\frac{\Gamma \left(\frac{k+1}{2}\right)}{\sqrt{\pi }}.$$

So finally:

A≤2πN∑k=0N−1N−1kCBf(t)N−k−12m1−2αB2f(t)k+1Γk+12ΓN2.

**Lemma** **3.** *If N≥6, then:*

$$\max_{k=0..N-1}\frac{\Gamma \left(\frac{k+1}{2}\right)}{\Gamma \left(\frac{N}{2}\right)}=1.$$

**Proof.**  As precised in the introduction of Srinivasan and Zvengrowski [24], Gauss [25] (page 147) proved that on the interval $\left[x_{0},+\infty \right)$ where $x_{0}\in \left[1.46,1.47\right]$, Γ is a monotonic increasing function. So, for $N-1\geq k\geq 2,\Gamma \left(\frac{k+1}{2}\right)\leq \Gamma \left(\frac{N}{2}\right)$. And because $\Gamma \left(1/2\right)=\sqrt{\pi },\Gamma \left(1\right)=1$, we have:

$$\max_{k=0..N-1}\frac{\Gamma \left(\frac{k+1}{2}\right)}{\Gamma \left(\frac{N}{2}\right)}=\max\left(\frac{\sqrt{\pi }}{\Gamma \left(\frac{N}{2}\right)},\frac{\Gamma \left(\frac{N-1+1}{2}\right)}{\Gamma \left(\frac{N}{2}\right)}\right)=\max\left(\frac{\sqrt{\pi }}{\Gamma \left(\frac{N}{2}\right)},1\right)$$

Because N≥6, and Γ is monotone and increasing on [3;+∞], we have $\Gamma \left(N/2\right)\geq \Gamma \left(3\right)\geq \sqrt{\pi }$. Hence the result. □

Using Lemma 3 allows us to write:

A≤2πN∑k=0N−1N−1kCBf(t)N−k−12m1−2αB2f(t)k+1.

We recall that $\sigma^{2}=t\frac{m^{1-2\alpha }}{B^{2}}$ and $f\left(t\right)=\frac{1-t}{t}$. Then we can write:

A≤2πN∑k=0N−1N−1kCBf(t)N−k−12σ21−tk+1.

We now conclude with the final bound on ξ:

ξ≤expC22m1−2αf(t)1+f(t)1(2πσ2)NA≤expC22m1−2αf(t)1+f(t)1(2πσ2)N2πN∑k=0N−1N−1kCBf(t)N−k−12σ21−tk+1≤2expC22m1−2αf(t)1+f(t)∑k=0N−1N−1kCBf(t)N−k−111−tk+1B22tm1−2αN−k−1≤2expC22m1−2αf(t)1+f(t)∑k=0N−1N−1kCt(1−t)2m1−2αN−k−111−tk+1≤2expC22m1−2αf(t)1+f(t)1−tN∑k=0N−1N−1kC2f(t)m1−2αN−k−1≤2expC22m1−2αf(t)1+f(t)1−tN1+C2f(t)m1−2αN−1.

This completes the proof of Proposition 3. □

**Proof** **of** **Theorem 5.** We combine Theorem 3 with Proposition 3. We also upper-bound N−1 by *N*. □

## Data Availability

Not applicable.
